# “High blood pressure comes from thinking too much”: Understandings of illness among couples living with cardiometabolic disorders and HIV in Malawi

**DOI:** 10.1371/journal.pone.0296473

**Published:** 2023-12-28

**Authors:** Jane Jere, Allison Ruark, Julie T. Bidwell, Rita M. Butterfield, Torsten B. Neilands, Sheri D. Weiser, Nancy Mulauzi, James Mkandawire, Amy A. Conroy

**Affiliations:** 1 School of Public Health, University of California Berkeley, Berkeley, California, United States of America; 2 Wheaton College, Biological and Health Sciences, Wheaton, IL, United States of America; 3 Betty Irene Moore School of Nursing, University of California Davis, Sacramento, California, United States of America; 4 Center for AIDS Prevention Studies, University of California San Francisco, San Francisco, CA, United States of America; 5 Division of HIV, Infectious Disease and Global Medicine, University of California San Francisco, San Francisco, CA, United States of America; 6 Invest in Knowledge, Zomba, Malawi; Duke University Medical Center: Duke University Hospital, UNITED STATES

## Abstract

Cardiometabolic disorders (CMD) such as hypertension and diabetes are increasingly prevalent in sub-Saharan Africa, placing people living with HIV at risk for cardiovascular disease and threatening the success of HIV care. Spouses are often the primary caregivers for people living with CMD, and understanding patients’ and partners’ conceptions of CMD could inform care. We conducted semi-structured interviews with 25 couples having a partner living with HIV and either hypertension or diabetes. Couples were recruited from HIV clinics in Malawi and were interviewed on beliefs around symptoms, causation, prevention, and treatment for CMD. Data were analyzed at the individual and dyadic levels using framework analysis and Kleinman’s theory of explanatory models as a lens. On average, participants were 51 years old and married for 21 years. Approximately 57%, 14%, and 80% had hypertension, diabetes, and HIV. Couples endorsed a combination of biomedical explanatory models (beliefs around physical and mental health) and traditional explanatory models (beliefs around religion and natural remedies), although tended to emphasize the biomedical model. Half of couples believed stress was the main cause of hypertension. For diabetes, diet was believed to be a common cause. In terms of prevention, dietary changes and physical activity were most frequently mentioned. For disease management, medication adherence and diet modifications were emphasized, with some couples also supporting herbal remedies, stress reduction, and faith in God as strategies. Participants were generally more concerned about CMD than HIV due to poor access to CMD medications and beliefs that CMD could lead to sudden death. Within couples, partners often held many of the same beliefs but diverged around which etiological or preventive factors were most important (e.g., stress versus diet) and the best diet for CMD. Health education programs should involve primary partners to build knowledge of CMD and address overlap with HIV, and reinforce accurate information on lifestyle factors for the prevention and treatment of CMD.

## Introduction

Low-income countries face a high burden of non-communicable diseases and are projected to see a significant increase in premature deaths and disability, particularly in sub-Saharan Africa [1]. People living with HIV are at increased risk for cardiometabolic disorders (CMD) such as hypertension and diabetes due to inflammation caused by HIV, and exposure to antiretroviral therapy (ART), as well as traditional risk factors such as family history, age, high body mass index, sedentary lifestyle, alcohol use, and smoking [2–9]. HIV-associated cardiovascular disease has tripled globally over the past 2 decades and accounts for 2.6 million disability-adjusted life-years per year, with a significant burden in sub-Saharan Africa [10]. In addition, people living with HIV and co-morbid conditions are less likely to start and remain on ART [11,12], further exacerbating CMD risk.

In Malawi, the disease prevalence in the general population ranges from 16% to 33% for hypertension and from 2% to 6% for diabetes [13,14]. Among those receiving HIV care in Malawi, approximately one-quarter of patients have hypertension and/or diabetes [15]. A study of Malawian adults living with HIV and hypertension found that only 19% of patients on antihypertensive medication achieved blood pressure control [16]. Further, another study of Malawian adults with HIV on ART found structural heart damage (e.g., left ventricular hypertrophy) in 13% of participants, and that structural abnormalities were significantly associated with uncontrolled hypertension [17]. These findings highlight a missed opportunity for prevention, early detection, and treatment for CMD among people with HIV, who are at high risk for developing long-term cardiovascular complications.

When developing effective prevention and treatment programs, it is imperative to consider how individuals’ explanations and perceptions about health and illness affect their lifestyle behaviors, adherence to treatment plans, and health outcomes [18]. According to Kleinman’s theory of explanatory models [18], patients hold beliefs about disease etiology, onset of symptoms, pathophysiology, course of illness (including disease severity), and treatment. These explanatory models are shaped by personal, familial, social, and cultural beliefs, education, religion, and past experiences with illness and healthcare [18]. Patients and their caregivers may view illness quite differently from healthcare providers, and it is important that providers understand these differences when providing patient education and clinical care. Such understanding is particularly critical for chronic diseases like CMDs, which require long-term medical management and the adoption of multiple behaviors to achieve disease control. Indeed, a systematic review of health beliefs in patients with chronic conditions (including hypertension and diabetes) showed associations between personal perceptions about illness and religious/spiritual/cultural beliefs, and medication adherence [19].

Although research has been conducted on explanatory models for single diseases like diabetes [20–22], hypertension [23–25], and HIV [26–29] in resource-limited settings, less research has focused on multiple co-occurring conditions, such as CMD and HIV. In Kenya, Malawi, and Tanzania, qualitative research revealed limited understanding and varied beliefs regarding hypertension among people living with HIV [30–32]. Regarding etiology, worrying about one’s HIV status and being stigmatized due to HIV status was believed to lead to hypertension. With regards to the course of illness, hypertension was widely perceived as more severe and dangerous than HIV [31–34]. Concerning treatment, studies also found that adherence to antihypertensive medications was lower than for ART because of sporadic drug availability, medication costs, use of herbs, and beliefs that medications could be stopped if symptoms go away [31–34]. Less research has examined beliefs about CMD among people living with HIV in sub-Saharan Africa, although studies have revealed limited knowledge about diabetes in the general population [35–38]. In Uganda, a study found that people perceived diabetes as a severe disease but they did not seriously consider the risk factors and behaviors needed to prevent the disease [39].

In settings with inadequate infrastructure to screen and manage CMD, the majority of caregiving for chronic disease occurs at home with the help of spouses or relatives [40,41]. Similarly, intimate partners or spouses are often the primary caregivers for people living with HIV in sub-Saharan Africa and provide a large burden of support to maintain health and well-being [42,43]. Research on cardiac health and diabetes in U.S. couples has found that spouses’ health beliefs, and the level of agreement between patient and spouse on these beliefs, can influence the support provided by the partner and the patient’s adherence to medical advice [44–46]. Similarly, established theories of couple and family management of chronic illness place shared understanding or appraisal of illness as an important upstream determinant of health behaviors, adjustment to living with illness, and/or health outcomes [47–49]. Importantly, family members or caregivers can impact patient adherence to care and disease management and may hold explanatory models that differ from those of the patient [18]. For instance, if a spouse has less knowledge and a different comprehension of the disease compared to the patient who receives information from their healthcare provider, this difference in understanding can lead to varying lifestyle habits and conflicting beliefs about the patient’s condition, potentially making the patient feel less supported [45]. Identifying areas of alignment and divergence in health beliefs between patients, caregivers, and providers may aid in developing approaches that involve families and particularly spouses to support disease management. However, to date, no research with African populations has explored spouses’ understandings of CMD and HIV, and relationship-level understanding of illness remains a critical gap that is impeding intervention efforts.

To address this gap, the present study aims to categorize and describe the explanatory models of CMD held by persons living with HIV and CMD (hypertension and/or diabetes) and their primary partners in southern Malawi. To address this aim, we expand explanatory models as theorized by Kleinman [18] to the context of multimorbidity (i.e., co-occurring HIV and CMD). We also address the etiology, symptoms, prevention, and treatment of HIV and CMD as a first step towards developing family-based health promotion interventions to improve lifestyle behaviors and treatment adherence for persons with HIV/CMD and their family care partners.

## Methods

### Study setting

Located in the southern region of Malawi, the Zomba district has a substantial HIV/AIDS prevalence, affecting nearly one in ten men and one in five women [50]. In addition, the prevalence of hypertension and/or diabetes in HIV clinics in Zomba is 26.6% [15]. The provision of hypertension and diabetes care follows the clinical guidelines set by the Malawi Ministry of Health [51]. Drugs for HIV are generally free and available, whereas medications for diabetes and hypertension are free only when in-stock at government-supported clinics.

### Study sample and recruitment

Fifty individuals comprising 25 couples were recruited from three facilities that serve approximately 19,000 persons living with HIV, and represent a diverse range of geographic and social contexts. To be eligible, participants had to be 18 years or older and married or cohabiting for at least 6 months. Each couple had to include one partner (referred to as the “index patient” for clarity) who was diagnosed with HIV and either hypertension or diabetes and who had disclosed their HIV and CMD status to their partner. Recruitment of index patients took place during their HIV clinic appointments, with research assistants announcing the study during the health information sessions in the waiting rooms. Research assistants, working in male-female pairs, approached potential participants, screened for eligibility, and if eligible, provided information cards to give to the primary partner. Research assistants would later contact and speak privately to the primary partner on the phone to assess their eligibility and willingness to take part in the study. Some diabetes and hypertension patients were screened and referred by the nurses in accordance with the clinical guidelines for CMD [51]. Partners who collected the index patients’ HIV medications were also approached and given information cards about the study, and then research assistants later contacted the index patient on the phone to assess eligibility. Once both partners were deemed eligible and willing to participate, research assistants scheduled the first appointment for the couple at the HIV clinic. During the appointment, the couple’s eligibility was confirmed, both partners gave written informed consent in separate locations, and an interview and short survey were conducted. Participants were reachable by phone. The research team adhered to established recruitment and follow-up procedures, making three contact attempts at different times and on different days to account for situations where phones might be out of network or uncharged.

### Study procedures and data collection

Semi-structured interviews were conducted in Chichewa by trained interviewers who were matched to the gender of the participants. Partners were interviewed separately in private areas to ensure confidentiality and independent responses, and to minimize bias. The interviews lasted between 60–90 minutes and were tailored to the CMD of the index patient, either hypertension or diabetes. If the patient had both conditions, questions were focused on diabetes due to its lower prevalence. The partner version of the guide focused questions on the index patient’s conditions. Both partners were asked questions according to domains of Kleinman’s theory of explanatory models [18] including symptomology, etiology, prevention, and disease management, as well as beliefs about the intersection of HIV and CMD. Examples of questions asked were “How do people get hypertension or diabetes?”, “What are the symptoms of hypertension or diabetes?”, “What can people do to prevent getting hypertension or diabetes?”, “What are some medicines that are used to treat hypertension or diabetes?”, and “What other ways can people manage their hypertension or diabetes besides medications?” To assess how participants compared and contrasted CMD with HIV, we asked: “How would you describe the relationship between HIV and hypertension (or diabetes)? Does one cause the other? Does one disease affect the other?”

### Data analysis

The interviews were audio-recorded, transcribed, and translated from Chichewa to English by research assistants who are fluent in both languages. We conducted an individual-level analysis and dyadic-level analysis using a framework analysis approach, which allows for comparative analysis of themes and cases by utilizing data matrices [52]. We read and reviewed the interview transcript pairs, created summary tables, and organized the data by couples and by the larger categories from Kleinman [18] that were derived from the interview guide (e.g., “beliefs about symptoms”, “beliefs about treatment”). We compared the responses within and between couples by including raw quotes from each partner and then summarizing all the participants’ responses by category and noting any agreements or disagreements between partners (e.g., wife believes diet causes hypertension; husband believes the causes are diet and stress). Within these categories, the main themes were selected based on the frequency in which they came up and by their intensity (i.e., not necessarily common, but important or unexpected) [53]. We also coded for the broad types of explanatory models that emerged inductively from the data as other researchers have done when applying Kleinman’s model [54].

The research team, which included representation from Malawi and other African settings, met regularly to discuss the findings using the summary tables and the themes that emerged.

### Ethics approval

This study received ethical approval from the National Health Science Research Committee in Malawi and the Human Research Protection Program at the University of California San Francisco. All participants provided written consent.

## Results

### Sample characteristics

Participants were married or cohabitating couples with a mean age of 51 years (range: 37–66) and mean relationship duration of 20.8 years (range: 4–40). Most participants were Christian (88%), and a minority were Muslim (12%). Participants were recruited from a district hospital clinic (56%), a rural private community hospital (36%), and a peri-urban public facility (8%). The majority (80%) of participants were living with HIV, and of those, all were on ART. Fifty-seven percent had hypertension and 85% of those were on antihypertensive medications. Fourteen percent had diabetes and of those, 86% were on oral medications for diabetes (none were on insulin). The characteristics of the study participants are displayed in Table 1.

**Table 1:** Demographics and disease status of participants.

	Index Patient (N = 25)		Partner (N = 25)		Total (N = 50)	
	% (N)	mean (SD)	% (N)	mean (SD)	% (N)	mean (SD)
Sex						
Female	52% (13)		48% (12)			
Male	48% (12)		52% (12)			
Age in years		52.2 (6.4)		50.4 (8.3)		51.3 (5.8)
Relationship duration (years)						20.8 (12.7)
Primary school education or less	72% (18)		64% (16)		68% (34)	
Religion						
Christian	88% (22)		88% (22)		88% (44)	
Muslim	12% (3)		12% (3)		12% (6)	
Recruitment site for index patient						
Urban district hospital					56% (28)	
Rural community hospital					36% (18)	
Peri-urban health center					8% (4)	
Living with HIV	100% (25)		60% (15)		80% (40)	
Time since diagnosis (years)		10.5 (5.4)		9.3 (6.0)		10.3 (5.1)
Currently taking medication	100% (25)		100% (15)		100% (40)	
Never/rarely missed pills, past week	96% (24)		93% (14)		95% (38)	
Living with HTN	84% (21)		24% (6)		54% (27)	
Time since diagnosis (years)		6.9 (6.0)		6.5 (4.8)		6.7 (5.7)
Currently taking medication	90.5% (19)		66.7% (4)		85% (23)	
Never/rarely missed pills, past week	84% (16)		75% (3)		83% (19)	
Living with DM	20% (5)		8% (2)		14% (7)	
Time since diagnosis (years)		3.7 (5.0)		7.9 (7.2)		4.9 (5.4)
Currently taking medication	100% (5)		50% (1)		86% (6)	
Never/rarely missed pills, past week	100% (1)		100% (5)		100% (6)	

### Overarching explanatory models

In their narratives, participants described the etiology, symptoms, disease course, prevention, treatment, and disease management of CMD. Table 2 outlines the common themes that emerged under each category with representative quotes from couples. Based on the themes that emerged in the data, we grouped explanatory models into two main categories: biomedical and traditional/cultural models. Biomedical explanatory models consisted of beliefs around physical health (e.g., lifestyle factors, prescribed medications) and mental health (e.g., stress, depression). Although other studies have considered stress or mental health as alternatives to biomedicine, we consider that beliefs about stress as a cause of CMD are consistent with biomedical research linking stress to CMD and cardiovascular disease [55–60]. We categorized beliefs as falling under a traditional/cultural explanatory model when grounded in religious beliefs and beliefs in natural medicine (e.g., herbs, traditional medicine) as a form of healing. Participants sometimes subscribed to a combination of explanatory models, both biomedical and traditional, sometimes giving more weight to one model over the other.

**Table 2:** Emerging themes and supportive quotes from couples living with cardiometabolic disorders and HIV based on Kleinman’s theory of explanatory models.

**Themes and supportive quotes from couples**	
***Etiology beliefs***	
Couple #1: Hypertension, female patient	“Some people say because of stress, that’s how they get hypertension”. *(Female index patient with HIV and hypertension)* “BP occurs because of thinking too much…the heart rate becomes very fast, and the heart is never calm…Most of the time this disease occurs because of how you speak to an individual or maybe how the person is living while depressed.” (*Her husband*, *HIV negative with no CMD*)
Couple #2: Diabetes, male patient	“People get this disease by not doing physical exercises…eating food like meat frequently.” (*Male index patient with HIV*, *diabetes*, *and hypertension*) “Maybe some food which we eat…like cabbage. I hear that when it has been planted, they do apply different types of pesticides. Cabbage leaves are always tight so sometimes the pesticides are still inside so even if you wash the outside, you still cut it together with the pesticides. I believe that maybe it was food that caused his sugars to be high…he liked eating foods containing sugar like sugarcane, bananas, and sweet potatoes.” (*His wife*, *HIV positive with no CMD*)
***Disease course and severity beliefs***	
Couple #3: Hypertension, male patient	“Hypertension is the one that proves to be difficult in taking care of [more difficult than HIV] …at times because somebody spoke to an individual and the particular individual is thinking about it too much [meaning stress]. The blood flow tends to get faster…maybe when they’ve been disappointed with something”. (*Male index patient with HIV and hypertension*) “Hypertension can kill you instantly even if one is taking the medicine and is not following other advice then there is nothing that can be done… at any time we can hear that the person is dead, while with HIV, there are some control measures.” (*His wife without HIV or CMD*)
***Symptomology beliefs***	
Couple #4: Hypertension, female patient	“Hypertension starts with headache, heart beats fast, when the heart is beating, it hurts, it stings very much, so when you go to the hospital after diagnosis, they find your blood pressure high. Sometimes you experience darkness in your eyes, dizziness, you feel like collapsing.” (*Female index patient*, *HIV positive with hypertension)* “The signs for me to know that she had hypertension is that she easily loses her temper. And sometimes when we disagree… she easily gets angrier than me. So, for me also when a person has done something which is not good for me, my heart flies off more than it used to be before I was diagnosed with hypertension.” (*Her husband*, *HIV negative with hypertension*)
Couple #5: Hypertension, male patient	“Firstly, frequent urination, the inside of the mouth gets dry, headache, the body gets wrinkles due to dehydration, short sight, you cannot see far. (*Male index patient*, *HIV positive with diabetes*) “…frequent urination, the heart beats fast…there is a lot” (*His wife*, *HIV negative and no CMD*)
***Prevention beliefs***	
Couple #6: Hypertension, male patient	“For someone who doesn’t have hypertension, they can prevent it through doing physical exercises like running and also doing heavy work…that way the body will be healthy, and the person will be able to avoid getting hypertension.” (*Male index patient with HIV and hypertension*) “Communicating peacefully…when a person just comes and talks to me in a shocking way, I see that my heart starts to beat fast, and the BP goes up”. (*His wife with HIV and hypertension*)
Couple #7: Diabetes, male patient	“People should eat more vegetables and also drink more water; they should also do physical exercises [to prevent diabetes]” (*Male index patient with HIV*, *diabetes*, *and hypertension*) “I believe that to prevent diabetes…eat foods of low sugar levels…don’t eat food with starch. I should say stop eating rice, groundnuts, sweet potatoes or even cassava.” (*His wife with HIV and no CMD*)
***Treatment and disease management beliefs***	
Couple #8: Hypertension, male patient	“Reduce fatty foods and do exercises habitually… salt needs to be reduced…taking medicine just weakens the disease.” (*Male index patient with HIV and hypertension*) “There isn’t real medication [which cures hypertension] but we just take medication for the rest of our lives…but not that they completely cure hypertension, no”. (*His wife with hypertension and no HIV*)
Couple #9: Diabetes, male patient	“To weaken diabetes, it’s by following guidelines. Firstly, take medication, secondly, avoid taking sugary food. You should be eating meals that are recommended for the condition to gain necessary energy”. (*Male index patient with HIV and diabetes*) “On the part of treating, I think it is just following instructions from the doctors and taking the medication for diabetes. I feel like it could help you”. (*His wife without HIV or CMD*)

### Etiology beliefs

More than half of the couples with hypertension adopted a biomedical explanatory model with a focus on mental health. They attributed their illness to “thinking too much” due to stress, which was described vaguely as “depression”, “the heart is not calm”, or feeling “restless” (see Couple #1 in Table 2). Some participants described stressful situations related to interpersonal relationships as causes for hypertension such as “the marriage is not going well”, “quarreling with a friend”, “someone recently died”, “disappointing each other”, and “living in an environment that is not peaceful”. Most of the participants who believed that stress was a cause of hypertension were index patients, but 23% of partners also mentioned stress. Participants seemed to come to this causation belief through personal experiences with people they knew to be stressed, or what they heard in their community rather than from healthcare providers. Of note, stress was not mentioned as a cause of diabetes.

In addition to a mental health model, index patients and their partners also adopted models focused on physical health and attributed hypertension to various dietary factors, such as high salt intake, excess fat, and sugar, certain meats, certain vegetables, cooking oil, pesticides on food, sour foods, and tea. In some cases, couples had more nuanced views on the type of diet that caused hypertension (such as Couple #2 in Table 2). A few participants attributed hypertension to aging. Regarding diabetes, commonly mentioned causes were the consumption of sugary drinks or food, including tea, followed by genetics and aging. A few participants said that they did not know what caused diabetes and one participant thought that viruses could cause diabetes. Physical inactivity and obesity were not identified as causes of hypertension, and only one participant mentioned that physical inactivity can cause diabetes.

HIV was not discussed as a potential cause of hypertension or diabetes, although a few participants suggested a possible link through the immune impact of HIV or how the stress of living with HIV over many years could lead to the development of hypertension. One partner believed that ART directly caused her husband’s hypertension because, in her view, he was diagnosed with hypertension once he started taking HIV medication; however, her husband did not mention the same belief.

### Symptoms

Couples reported a varied range of physical and mental symptoms of hypertension and diabetes, with only one person stating that hypertension can be asymptomatic. Hypertension symptoms were based on experiences as patients, observations as partners, or what participants had heard from others. One-third reported a fast heart rate (“heart racing”) as a symptom and a few others described “heart pain” or “heart pricking” as symptoms. Other reported signs and symptoms varied widely among participants and included fever, sweating, headaches, body pains, difficulty breathing or “panting,” poor vision, “seeing darkness”, dizziness, weight gain, numbness, and loss of appetite (see Couple #4 in Table 2). Stroke was also mentioned alongside signs and symptoms as a sequela of hypertension. Secondary to the emphasis on physical signs and symptoms, some participants and partners also believed in mental health-related symptoms, stating that being easily stressed, short-tempered (see Couple #4 in Table 2), or “thinking a lot” was indicative of hypertension. Hypertension symptoms were sometimes compared with those of HIV, for example, one female patient asserted that both illnesses share similar symptoms. She seemed to equate the stress response of an HIV diagnosis to a similar set of symptoms for hypertension, stating: **“**The symptoms are the same because when you are diagnosed with HIV…you feel your heart racing, the body gets hot, the head hurts. Similarly, when you are suffering from hypertension your head hurts, your body feels hot, and the heart also races.”

For diabetes, most patients and their partners mentioned frequent urination, weakness, and vision changes as the main symptoms (see Couple #5 in Table 2). Other signs and symptoms included weight loss, drinking a lot of water, wrinkles on the skin, fainting, and other skin-related symptoms such as “blisters in the vagina” and poor wound healing.

### Disease course and severity: Comparing HIV and CMD

Around two-thirds of couples agreed that the index patient’s hypertension or diabetes was more serious than their HIV (see Couple #3 in Table 2); the remainder of couples had discrepant views, including a few couples with a partner who was unconcerned about each condition or worried equally about both. For most participants, the threat of hypertension was viewed as more concerning than that of HIV, with the general belief that HIV was more manageable, but hypertension could cause sudden death. There were beliefs that people with HIV generally outlive those with hypertension or diabetes, who could suddenly collapse without any signs.

Other explanations for the severity of hypertension had to do with the belief that missing pills for hypertension was deadly: “With AIDS, even if they can skip medication for some days, they can still live. While hypertension, if you skip medication for some days… you are likely to be in danger.” Similar sentiments were noted about diabetes in reference to HIV, with participants suggesting that when sugar levels are too high, a person can die unexpectedly (“this disease comes with force”) or become very weak and then fall unconscious. Most people noted that HIV medications were reliable, and people rarely died if taking ART; however, medications for hypertension and diabetes were believed to be insufficient for controlling the disease or preventing death, and thus, could not always be relied on: “Hypertension is the one that’s really scary. …what’s happening in most cases is that they are taking medication but it’s not helping them. Even those taking medication are still dying.”

Hypertension and diabetes were also understood as being harder to manage due to symptoms waxing and waning over time, and because disease control depended on more than just taking medication, as with HIV. People expressed frustration that hypertension and diabetes were difficult to manage because of factors beyond their control, such as exposure to stress and being unable to afford the recommended foods, medications, and devices necessary to monitor blood pressure and sugar levels. Unstable access to CMD medications is a major challenge in settings like Malawi, with structural barriers and clinical-level barriers such as medication and staff shortages, clinic wait time, as well as transportation costs to and from the clinics [34]. The uncertainty of hypertension and diabetes was also overwhelming for partners, some of whom expressed worries about taking care of the children if a spouse were to die or caring for a disabled spouse after a stroke.

### Prevention beliefs

Most couples believed that changes in diet and physical activity could prevent hypertension and diabetes. Salt reduction was noted as a preventive measure, and some participants suggested sugar reduction, even for hypertension. Participants described how sugar or salt can increase one’s heart rate, which was believed to cause “BP” (meaning hypertension). Exercise, like jogging or running, smoking cessation, and alcohol reduction were mentioned as ways to prevent hypertension and diabetes (see Couple #6 in Table 2). For diabetes prevention, the majority of participants stated that dietary changes, exercise, and medications were strategies for prevention. Some participants suggested avoiding certain vegetables, certain meats, cooking oils, rice, other starchy foods, tea, and nuts, and reducing sugar intake (see Couple #7 in Table 2). Similar to causation beliefs, stress reduction was frequently mentioned as a method to prevent hypertension, with suggestions like "avoiding thinking too much", “speaking calmly to a partner”, and "living peacefully" (see Couple #6 in Table 2). In a few cases, traditional explanatory models were invoked by a few participants, with praying being noted as a prevention strategy and some believing that God could keep a person healthy and free of CMD. One female participant with HIV and hypertension believed that condoms would prevent hypertension, although she was the only participant to link sexual risk with CMD.

In some couples, partners had different views on what they believed was most important for prevention. For example, one female participant with hypertension mentioned that medications could prevent hypertension, whereas her husband without CMD said: “the individual has to avoid thinking too much…they should remove all the stress they have and if they do, things will go well.”

### Treatment beliefs

All couples emphasized the importance of taking medications as prescribed for hypertension management, despite some concern around taking them “for their entire life”. While a minority of participants believed medications could cure CMD, most understood they only controlled the disease (see Couple #8 in Table 2). Lifestyle modifications, such as dietary changes and exercise, were also emphasized for CMD management. Some suggested reducing oils, meat, and salt for hypertension management. Diabetes management strategies mentioned by participants included dietary changes such as sugar reduction and medication (see Couple #9 in Table 2).

Although less commonly mentioned in comparison to the biomedical approaches around physical health, some participants mentioned the importance of stress reduction, self-acceptance of the diagnosis, and faith in God for hypertension management. Participants and their partners commonly shared the belief that herbs, such as hibiscus, moringa, lemongrass, ginger, garlic, and onions, could be used for CMD management. Some had personal experience using these herbs to manage their CMD, and while others had only heard of traditional medicines like *mvunguti* (derived from the tree species *Kigelia africana)* for CMD management. Most participants had not consulted traditional healers due to conflicts with religious beliefs or perceptions that traditional healers were unsuitable for managing HIV and CMD, with some having done so, but then stopped after receiving their CMD diagnoses at healthcare facilities.

In most couples, partners were often congruent on general beliefs about medical and lifestyle management (with some nuanced differences such as agreement on diet type). However, there were some couples who seemed to diverge in the type of explanatory model invoked. For example, the husband of a wife living with hypertension believed that spirituality was more important than medications: “It’s not difficult to get cured and does not depend on medications because hypertension does not have medications (a cure). A person can take medications all his life, but if you would take God and put him close”, he also noted how he preferred that she take herbs that would cure her condition and “put an end to it”. His wife, the index patient, emphasized the importance of following the advice of the health providers, admitted that she had not tried herbs yet, and did not mention spirituality. In another case of a male participant living with hypertension, the wife stated: “I feel like the cure for this disease is that first, you should accept it…when you accept it and believe that this thing (hypertension) is not going to be with me, it is possible…the heavenly one (God) is the one who has all the possibility of helping you…I tell my husband that you should ask God to take this away from you”. Her husband, the index patient, believed in taking prescribed medication and following medical advice on lifestyle changes, indicating a discrepancy between partners’ explanatory models for treatment.

## Discussion

Against the backdrop of rising rates of chronic disease in sub-Saharan Africa with the potential to threaten the success of HIV care, we explored explanatory models of illness among couples living with CMD and HIV in Malawi. Using Kleinman’s theory, we described beliefs across the care continuum ranging from prevention to disease management. Given the critical role of relatives and primary partners for caregiving and disease management in this setting, our innovative approach captured the perspectives of patients living with CMD and their spouses. We found that patients and partners held a mix of biomedical models, which centered on physical and mental health, and traditional models, which included religious influences and herbal medicine. The biomedical model was the primary model adopted by participants, although within the same model, equal emphasis was placed on physical and mental health, particularly the role of stress in causation, prevention, and disease management. Participants were more likely to embrace a traditional model when it came to disease management as compared to symptomology or causation, which were almost always framed using a biomedical model. The synthesis of models is consistent with other studies on explanatory models of CMD from Western and non-western settings including Ethiopia [54,61,62], which found that people invoke a combination of Western biomedicine and traditional models to understand illness.

Participants’ reliance on a biomedical explanatory model could be related to experiences of living with HIV for many years and repeated exposure to the biomedical health care paradigm. Early work from the beginning of the AIDS epidemic in sub-Saharan Africa suggests that other types of traditional models [28,63–68] were commonly invoked to explain emerging infectious diseases. However, several decades of exposure to clinical care for HIV and more recently, widespread access to effective ART, has paved the way for use of biomedical explanatory models to understand newer forms of chronic disease in this region such as diabetes. In a few cases, participants relied heavily on their knowledge of HIV when understanding the causation and prevention of CMD, for example, one participant believed that HIV caused hypertension and using condoms could prevent hypertension. Although these cases were uncommon in our data, health implications are significant and require attention, with follow-up investigations needed to understand the prevalence and nuances of beliefs that more closely link CMD with direct experiences of HIV.

Despite having limited care services for CMD in this setting, primary partners were relatively knowledgeable about the role of diet and other lifestyle factors in prevention and management. Partners often expressed similar types of explanatory models as patients around physical and mental health, although some slightly diverged when it came to defining a heart-healthy diet or on specific lifestyle advice. This is encouraging news and suggests that couples are actively communicating with each other around CMD, and knowledge is being shared between partners. In other cases, partners both presented unique, but not necessarily contradictory, perspectives. for example, one partner focusing on diet and the other highlighting stress—illustrating a more comprehensive set of beliefs at the couple level that could impact support and disease management if summed together. Other studies have demonstrated how couple communication and knowledge-sharing influences HIV disease management [69–72], although the role of partners’ beliefs may depend on power dynamics and social control in terms of how much influence one partner has over another partner. Gender norms and different power dynamics between a couple can shape communication patterns and impact the negotiation of health-related behaviors, potentially limiting the less influential partner’s ability to assert their health beliefs and preferences [73,74].

In terms of the etiology of CMD, the belief that stress and the stress of interpersonal problems (e.g., death, conflict) were widely accepted by over half of the participants. Stress as a cause has been described in other qualitative studies from Malawi, Tanzania, and Ethiopia as an important component of explanatory models for hypertension and diabetes [33,54,69,75]. There were only a few cases of participants noting that HIV could lead to CMD through the stress of living with HIV or through exposure to ART, the latter of which is partially supported by research suggesting that ART use is associated with increased blood pressure [76,77]. A wide range of symptoms were noted by the couples despite hypertension and diabetes being largely asymptomatic conditions in the early stages [59,78–80]. Because blood pressure and diabetes screening are not always routinely offered, it is possible that CMD patients in this study had more severe cases of longstanding, uncontrolled hypertension, or diabetes, leading to symptomatic but undiagnosed sequelae (e.g., development of complications, comorbidities, etc.). It could also be that patients are basing their beliefs on experience with infectious diseases, and thus are attributing symptoms of viral disease to CMD. Indeed, one participant even mentioned how HIV and hypertension have similar symptoms (e.g., the body gets hot). In contrast, beliefs about symptoms of diabetes such as weight loss and frequent urination were largely consistent with biomedical knowledge [4,78,81].

Hypertension and diabetes were almost universally understood as being more severe and concerning than HIV because treatment for CMD was viewed as inaccessible and unreliable in contrast to ART. These understandings created feelings of uncertainty and worry that a partner could die suddenly and prematurely from CMD, likely without warning. This aligns with another qualitative study from Malawi in which participants viewed hypertension as concerning as HIV, primarily due to the inability to anticipate the health consequences of hypertension and access antihypertensive medications [34].

In terms of prevention, participants emphasized the importance of diet, physical activity, and reductions in alcohol and tobacco use. There were some suggestions among some couples that medication could prevent or even cure CMD and some couples were conflicted on the biomedical role of medications versus stress in preventing disease. A few participants used a traditional model to emphasize the role of spirituality in preventing disease. Many of the same lifestyle behaviors for prevention were described as strategies for disease management, in some cases aligning with Western guidelines for primary, secondary, and tertiary prevention. In addition, some people mentioned using herbs as supplements to biomedical care for CMD, whereas for others, there was some resistance around consulting traditional healers due to conflicting religious beliefs or concerns that traditional medicine was not appropriate. In some cases, religion and “faith in God” were believed to be curative for CMD and partners were not always in agreement on the relative importance of biomedicine versus spirituality as the primary method for disease management.

Together, findings suggest that health education around prevention, symptomology, and disease management for CMD should be a first step and priority. While stress reduction and mental health are important for overall well-being, there may be a need to shift the focus of messages to diet, exercise, and alcohol and smoking as primary risk factors for CMD and targets for secondary and tertiary prevention. At the same time, couples indicated high levels of stress and worries around CMD and thus screening for depression and anxiety could be considered if services were available to treat such conditions. Related to findings on symptomology, patient education should describe the signs of severe hypertension or diabetes with patients and their partners to help recognize early warning signs of danger and when to seek prompt medical care. Additionally, patients with hypertension should understand that signs and symptoms may be indicative of other complications or disease processes that may require clinical evaluation and treatment. Finally, as others have called for [34], there is an urgent need to strengthen healthcare services for HIV and CMD using lessons learned from successful HIV care programming and building trust in antihypertensives and other CMD medications by ensuring a reliable supply and good patient and family education. Additionally, this effort would involve universal screening/monitoring of blood pressure and blood glucose levels, along with subsequent provision of feedback to the patients. It is important to also consider conflicting explanatory models within couples when strengthening health services for CMD and education efforts. Clinicians could use Kleinman’s framework [18] to elicit patient and partner explanatory models of CMD to help tailor discussions of lifestyle changes and adherence to care within the couple’s own unique set of models. In accordance with the explanatory models’ approach, it may be best to meet and understand patients and partners where they are, rather than forcing a particular paradigm that does not align with their beliefs or to which it may be difficult or impossible to adhere. Moreover, in cases where partners have different explanatory models, it may be particularly important to find a common ground in beliefs and also address the influence of power imbalances in relationships (especially gendered power imbalances), which could interfere with successful disease management and couple adjustment to living with chronic illness. Health education for CMD should also be tailored to address couples’ concerns and influences around religion/spirituality and traditional healing. Finally, in alignment with prevailing theories of chronic illness management in couples and other types of care dyads [47–49], education efforts would likely benefit from involving both partners together in order to foster social support and dyadic management of disease outside of the clinic and prevent CMD among others in the family.

## Limitations

We aimed to enroll an equal number of hypertension and diabetes patients but had difficulties enrolling diabetes patients given the lower prevalence of the disease in the population. Thus, because our sample is more heavily weighted by hypertension beliefs, we may not have reached data saturation levels for diabetes. We were unable to assess the severity of the participants’ CMD, and thus our sample may not have captured the full range of disease(mild/severe), for example individuals with severe and poorly controlled diabetes may confront unique challenges in disease management and daily living which may influence the beliefs held by both the patient and their partner. Nevertheless, there were many common themes that emerged in the subset of couples with diabetes and we are relatively confident that we captured the range of beliefs and explanatory models. In addition, we recruited couples from HIV clinics who were actively involved in care, and thus our sample may be more skewed towards a biomedical model as compared to others without the experience of HIV or CMD. Primary prevention efforts of CMD will need to actively take into account the varying cultural/traditional perspectives of chronic disease, and potentially greater differences between family members’ explanatory models before programs can be effectively rolled out in communities.

## Conclusion

Couples adopted a synthesis of biomedical and traditional models to understand CMD and its relationship with HIV, with a strong emphasis on the biomedical paradigm. Couples were largely congruent in the overall type of explanatory model employed and its underlying beliefs; however, some couples differed in the nuances of these beliefs (e.g., diet type) or invoked very different explanatory models. The specificity of knowledge is important and should be addressed in future health education campaigns to meet couples where they are at and address both partners’ perspectives of CMD. This includes correcting misbeliefs and re-iterating correct knowledge around the role of diet, physical activity, and other lifestyle factors in CMD prevention and treatment. Education efforts should also involve primary partners to reinforce the same set of messages and knowledge and build social support. Mental health needs also should be considered for couples living with the stress of multiple health conditions such as HIV and CMD, and to improve couple coping with disease, health, and well-being.

## Supporting information

S1 File (DOCX)
